# Diffuse traumatic brain injury substantially alters plasma growth hormone in the juvenile rat

**DOI:** 10.1530/JOE-23-0157

**Published:** 2023-11-20

**Authors:** J Bryce Ortiz, Sebastian Tellez, Giri Rampal, Grant S Mannino, Nicole Couillard, Matias Mendez, Tabitha R F Green, Sean M Murphy, Rachel K Rowe

**Affiliations:** 1 Barrow Neurological Institute at Phoenix Children’s Hospital, Phoenix, Arizona, USA; 2 Department of Child Health, University of Arizona College of Medicine, Phoenix, Arizona, USA; 3 Arizona State University, School of Life Sciences, Tempe, Arizona, USA; 4 Department of Biology and Biochemistry, University of Bath, Bath, United Kingdom; 5 Department of Integrative Physiology, University of Colorado, Boulder, Colorado, USA; 6 Cumberland Biological and Ecological Researchers, Longmont, Colorado, USA

**Keywords:** concussion, growth hormone deficiency, somatostatin, puberty, development, pediatric

## Abstract

Traumatic brain injury (TBI) can damage the hypothalamus and cause improper activation of the growth hormone (GH) axis, leading to growth hormone deficiency (GHD). GHD is one of the most prevalent endocrinopathies following TBI in adults; however, the extent to which GHD affects juveniles remains understudied. We used postnatal day 17 rats (*n* = 83), which model the late infantile/toddler period, and assessed body weights, GH levels, and number of hypothalamic somatostatin neurons at acute (1, 7 days post injury (DPI)) and chronic (18, 25, 43 DPI) time points. We hypothesized that diffuse TBI would alter circulating GH levels because of damage to the hypothalamus, specifically somatostatin neurons. Data were analyzed with generalized linear and mixed effects models with fixed effects interactions between the injury and time. Despite similar growth rates over time with age, TBI rats weighed less than shams at 18 DPI (postnatal day 35; *P* = 0.03, standardized effect size [*d*] = 1.24), which is around the onset of puberty. Compared to shams, GH levels were lower in the TBI group during the acute period (*P* = 0.196; *d* = 12.3) but higher in the TBI group during the chronic period (*P* = 0.10; *d* = 52.1). Although not statistically significant, TBI-induced differences in GH had large standardized effect sizes, indicating biological significance. The mean number of hypothalamic somatostatin neurons (an inhibitor of GH) positively predicted GH levels in the hypothalamus but did not predict GH levels in the somatosensory cortex. Understanding TBI-induced alterations in the GH axis may identify therapeutic targets to improve the quality of life of pediatric survivors of TBI.

## Introduction

Annually, approximately 70 million people worldwide suffer from a traumatic brain injury (TBI) (Dewan *et al.* 2018), with 1.5 million of these individuals residing in the USA. Among the 1.5 million, around 50,000–60,000 are children (< 18 years) who require hospitalization, of which nearly 3000 do not survive (Peterson *et al.* 2021). TBI is a leading cause of mortality and morbidity in children (Faul *et al.* 2010, Dewan *et al.* 2016), and the effects of a TBI during childhood can produce lifelong consequences (Keenan & Bratton 2006). Individuals who experience a TBI during childhood are at increased risk for neurological and psychological disorders (Babikian *et al.* 2015, Ryan *et al.* 2016), drug and alcohol dependence (Jorge *et al.* 2005), and physical disabilities (Thurman 2016). Although the cause of TBI may vary among demographics and age groups, TBI in the pediatric population is primarily associated with falls (Dewan *et al.* 2016), accidents from daily activities, participation in sports (Prins *et al.* 2010), and domestic violence/child abuse (Rowe *et al.* 2021, Sayrs *et al.* 2022).

Despite the primary injury from a TBI being irreversible, subsequent injury processes occur in a delayed manner and may be responsive to medical management. The mechanical forces of TBI and the resultant secondary injury mechanisms lead to neuropathology and cellular damage to internal structures of the brain, including the hypothalamus and pituitary (Sundaram *et al.* 2013). Damage to these regions directly influences the endocrine system. Evidence suggests that endocrinopathies (i.e.disorders of the endocrine system), occur in as many as ~50% of pediatric patients with a history of TBI (Acerini *et al.* 2006, Rose & Auble 2012, Casano-Sancho *et al.* 2013, Reifschneider *et al.* 2015). Endocrinopathies are defined as long-lasting changes in the production, release, circulation, and/or regulation of hormones. Some studies have found that experimental TBI in adult rodents can lead to alterations in hormone release (Rowe *et al.* 2016*a*), which may be enduring (Greco *et al.* 2013). However, the physiological and functional changes that occur in juveniles due to TBI-induced endocrinopathies remains unknown.

In juveniles, activation of the growth hormone (GH) axis results in linear body growth and changes in body size and composition (Blakemore *et al.* 2010). This physical growth and increased height are linked to high levels of GH secretion. Injury to the hypothalamus can cause improper activation of the GH axis, leading to growth hormone deficiency (GHD) (Kgosidialwa *et al.* 2019). GHD places children at risk for delayed skeletal maturation, short height, psychosocial difficulties, and metabolic syndrome (Ibanez *et al.* 2000) in adulthood. Although GHD is the most common hormone deficiency in adults following TBI (Popovic 2005), the prevalence of juvenile GHD following TBI, and how GHD affects juveniles over the course of development, remains unclear. GH release is regulated by the hypothalamic peptides somatostatin and growth hormone releasing hormone (GHRH). Somatostatin is released from somatostatin-expressing neurons in the periventricular nucleus of the hypothalamus, whereas GHRH is released from GHRH-expressing neurons in the arcuate nucleus of the hypothalamus. TBI-induced alterations of neurons that regulate the release of GH have not been evaluated in juvenile rodents. However, GH alterations after TBI are of significant interest because, if untreated, GHD impairs the rehabilitation and recovery of TBI survivors (Kreber *et al.* 2016).

Clinical studies indicate that over a 1-year span patients who suffer a TBI are at a higher risk for developing an endocrine system disorder than those who have not sustained a TBI. We have previously shown that specific to the pediatric population, children with a TBI diagnosis have 3.22× the risk of a subsequent endocrine disorder diagnosis compared with the general pediatric population (Ortiz *et al.* 2019). Furthermore, we have reported the predominant endocrine disorder diagnoses following pediatric TBI is ‘precocious sexual development and puberty’, followed by, ‘pituitary dwarfism/GH deficiencies’ (Ortiz *et al.* 2019). We used these clinical observations to inform our translational research investigating GH-axis disruption following diffuse TBI in the juvenile rat. In the current study, we used postnatal day 17 rats, which model the late infantile/toddler period (Sengupta 2013, Picut *et al.* 2015), and assessed body weights, plasma GH levels, and mean number of hypothalamic somatostatin neurons at acute and chronic time points post injury. We hypothesized that diffuse TBI in juvenile rats would alter circulating GH levels because of damage to the hypothalamus, specifically somatostatin neurons. To improve personalized medicine, identifying the relationship between TBI and GH is necessary to understand how injury-induced damage may lead to the development and progression of GH alterations and chronic endocrinopathies.

## Materials and methods

### Rigor

All animal studies were conducted in accordance with the guidelines established by the Institutional Animal Care and Use Committee (IACUC) at the University of Arizona and the NIH guidelines for the care and use of laboratory animals. Studies are reported following the Animal Research: Reporting In Vivo Experiments (ARRIVE) guidelines (Kilkenny *et al.* 2010). Animals were randomly assigned to treatment groups and time points before the initiation of the study to ensure equal distribution of experimental conditions across all groups. Data collection stopped at predetermined endpoints based on days post injury (DPI) for each animal. Quantification of growth hormone levels and somatostatin neurons was performed by investigators blind to the experimental treatments. For data analyses, a total of 83 rats were used (sham *n* = 36; TBI *n* = 47). Predetermined exclusion criteria were excluding any rat that lost >20% of their body weight or had unmanageable pain. No rats in this study met those criteria, so none were excluded. Predetermined inclusion criteria included a righting reflex time >120 s and no breach of the dura. All plasma samples and slides were relabeled with a code by an investigator not associated with the study to ensure that investigators were blinded to the experimental conditions.

### Animals

**Figure 1:**
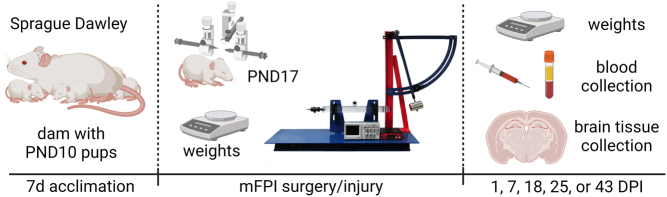
Study design. Postnatal day (PND) 10 rats were received with their dam and acclimated for 7 days. PND 17 rats were subjected to midline fluid percussion injury (mFPI) or control sham surgery. Body weights were taken at surgery, weekly post injury, and at the time of tissue collection. Blood and tissue samples were collected at 1, 7, 18, 25, or 43 days post injury (DPI).

Juvenile male Sprague Dawley rats (Envigo, Indianapolis, IN, USA) were used for all experiments. Rats were housed in a 12 h light:12 h darkness cycle at a constant temperature (23°C ± 2°C) with food and water available *ad libitum* according to the Association for Assessment and Accreditation of Laboratory Animal Care International guidelines. Rats were shipped with the dam at postnatal day 10 and were acclimated for a minimum of 1 week prior to experiments (Fig. 1). Weights and health conditions were monitored and documented throughout the experiment. Animal care and experiments were approved by the Institutional Animal Care and Use Committee (IACUC) at the University of Arizona (protocol 13-460). Group sizes for GH quantification and body weights were: 1 DPI sham *n* = 10; 1 DPI TBI *n* = 10; 7 DPI sham *n* = 7; 7 DPI TBI *n* = 10; 18 DPI sham *n* = 5; 18 DPI TBI *n* = 8; 25 DPI sham *n* = 5; 25 DPI TBI *n* = 9; 43 DPI sham *n* = 9; 43 DPI TBI *n* = 10. Group sizes for quantification of somatostatin neurons were: 1 DPI sham *n* = 5; 1 DPI TBI *n* = 5; 7 DPI sham *n* = 3; 7 DPI TBI *n* = 4; 43 DPI sham *n* = 4; 43 TBI sham *n* = 5.

### Midline fluid percussion injury

For surgery, all rats were administered 5% isoflurane in 100% oxygen for 5 min and then secured in a stereotaxic frame. Anesthetization was maintained with continuous isoflurane delivery at 2.5% via nosecone. A midline incision was made and a craniectomy (outer diameter 3mm) was trephined midway between bregma and lambda (Rowe *et al.* 2018). The skull was removed with care not to disrupt the dura or superior sagittal sinus underlying the craniectomy site. An injury hub prepared from the female portion of a Luer-Loc needle hub was fixed over the craniectomy using cyanoacrylate gel and methyl-methacrylate (Hygenic Corp., Akron, OH, USA). Post surgery, rats were placed on a heating pad and monitored until ambulatory.

Approximately 60–120 min after surgery, rats were subjected to midline fluid percussion injury (mFPI) with methods we have previously described for postnatal day 17 rats (Rowe *et al.* 2016*b*, 2018, Doust *et al.* 2021, Green *et al.* 2021). Rats were reanesthetized with 5% isoflurane in 100% oxygen delivered for 3 min. The hub assembly on the skull was filled with saline and attached to the FPI device (custom design and fabrication, Virginia Commonwealth University, Richmond, VA, USA). When a toe pinch withdrawal response was detected, the pendulum was released causing a fluid pulse directly onto the dura resulting in a moderate brain injury in all rats (1.5 atmospheres pressure (atm)) (Rowe *et al.* 2016*b*, 2018, Doust *et al.* 2021). Sham rats were connected to the device, but the pendulum was not released. Hubs were removed immediately after injury or sham injury and rats were monitored for apnea, righting reflex time (time from the initial impact until the rat spontaneously righted itself from a supine position), and a fencing response (Hosseini & Lifshitz 2009). After rats spontaneously righted, brains were inspected for herniation, hematomas, and integrity of the dura. Brain-injured rats included in this study had an average righting reflex time of 239 s, indicative of a mild to moderate injury (Rowe *et al.* 2016*b*, 2018, Green *et al.* 2021), and had no disruption to the underlying dura. Rats were reanesthetized and scalp incisions were cleaned with sterile saline and closed with sutures. Rats were placed in a heated recovery cage and monitored until ambulatory. Rats were rubbed with bedding from their home cage and returned to their dam until tissue collection (1 DPI) or until they were weaned at postnatal day 24. Rat welfare was evaluated and documented daily during postoperative care via physical examination.

### Tissue collection

Blood was collected via cardiac puncture at predetermined time points post injury (1, 7, 18, 25, 43 DPI; Fig. 1). GH secretion occurs in a pulsatile fashion and is influenced by the circadian rhythm, so all blood samples were collected at approximately the same time of day (zeitgeber 3–5; 09:00–11:00 h). Brains were collected from a subset of rats at 1, 7, and 43 DPI. Rats were injected (intraperitoneally) with Euthasol (0.002 mL/g, Patterson Veterinary, Greeley, CO, USA), and approximately 300 µL of blood were collected via cardiac blood draw using a needle and syringe coated in EDTA. Blood was transferred to an EDTA-coated tube and was centrifuged to collect plasma for each rat. Plasma samples were stored at −20°C until GH quantification. Immediately following blood collection, rats underwent transcardial perfusion with ice-cold 1× PBS followed by 4% paraformaldehyde (PFA). Brains were removed from the skull and drop fixed in 4% PFA for 24 h. For cryoprotection, brains were successive incubated in 15% and 30% sucrose, each for 24 h. Brains were frozen and cryosectioned in the coronal plane at 40 µm and mounted on superfrost slides and stored at −80°C.

### Immunohistochemistry

Antigen retrieval was performed using sodium citrate buffer (pH 6.0). Slides were then washed in 1× PBS. Hydrophobic barrier pen was used around the perimeter of the slide, and slides were placed in a humidity chamber. Slides were incubated in blocking solution (4% normal donkey serum [NDS], 0.4% Triton 100 in 1× PBS) for 60 min. Following blocking, slides were incubated in primary antibody solution (rabbit anti-somatostain-14; Peninsula Laboratories Inc. cat #T-4103; at 1:1000 concentration in 1% NDS, 0.1% Triton 100 in 1× PBS) overnight at 4ºC. Slides were then washed in 1× PBS + 0.1% Tween 20. Slides were incubated in secondary antibody solution (biotinylated horse anti-rabbit IgG (H + L); vector BA-1100; at 1:250 concentration in 4% NDS and 0.4% Triton 100 in 1× PBS) for 60 min. Slides were washed in 1× PBS + 0.1% Tween 20. Endogenous peroxidases were blocked in 200 mL 1× PBS + 8 mL H_2_O_2_ for 30 min. After washing in 1× PBS + 0.1% Tween 20, slides were incubated in avidin–biotin complex (ABC) solution (Vectastain ABC kit PK-6100) for 30 min. Slides were washed in PBS + 0.1% Tween 20 and then incubated in DAB solution (from Vector DAB peroxidase substrate kit SK-4100) for 10 min and, following this, slides were immediately placed in water. The brain tissue was dehydrated in ethanol (70%, 90%, and 100%), cleared with citrosolve, and coverslips were added using dibutylphthalate polystyrene xylene mounting medium.

### Imaging and analysis

Images of stained tissue were taken using Zeiss Imager A2 microscope via AxioCam MRc5 digital camera and Neurolucida 360 software. For somatostatin neurons, quantification was completed from images ranging from −1.65 to −3.60 in reference to Bregma using a modified version of unbiased stereology. The regions of interest (ROI) were the medial preoptic nucleus, from rostral to the decussation of the anterior commissure, through the caudal end of the decussation of the anterior commissure (Peterfi *et al.* 2010), and the primary somatosensory cortex. Three investigators blinded to experimental conditions counted all stained neurons in six to nine images per rat. Cell counts were performed using ImageJ software to manually label each cell using the cell counter. These counts were averaged across sections per rat and across investigators to determine the mean number of somatostatin neurons per image per rat.

### Quantification of growth hormone

Plasma GH levels were quantified using enzyme-linked immunosorbent assay (ELISA) kits (Thermo Fisher Scientific, cat #KRC5311). GH samples were run in triplicates following the manufacturer’s instructions.

### Statistical analyses

We used generalized linear models to investigate differences in righting reflex times, apnea times, body weights, growth hormone levels, and number of neurons between sham and injured rats across time (DPI). Righting reflex times and number of neurons were overdispersed counts, so we specified negative-binomial error distributions in all models that contained those outcome measures (Hilbe 2014). Apnea times were counts that exhibited zero-inflation but were not under or overdispersed; therefore, we specified a zero-inflated Poisson error distribution in the apnea model (Hilbe 2014). Growth hormone levels were zero-truncated, severely right-skewed, and positive-only values; thus, we specified Gamma error distributions in all growth hormone outcome models (Bateson 2009). In contrast, despite being zero-truncated and positive-only values, exploratory analyses indicated that body weights were approximately normally distributed; therefore, we specified Gaussian error distributions in all body weight outcome models. We fit two generalized linear models to each outcome, one in which days post injury was the only fixed effect predictor and another model with a two-way interaction between the treatment (i.e. sham versus injured) and postnatal age fixed effects.

We were also interested in investigating whether the rate of increase (i.e. growth rate, *λ*) of body weight that naturally occurs with increasing pubescent rat age differed between sham and injury. To test this and quantify the rate of increase, we fit a generalized linear mixed effects model with random intercepts for individual rats (Bolker *et al.* 2009). We suspected that the relationship between body weight and postnatal age would be nonlinear (Garcia *et al.* 2000), which we modeled with a four-knot basis spline on postnatal days (Perperoglou *et al.* 2019). Additionally, we were interested in whether the number of somatostatin neurons could be used as a viable predictor of growth hormone levels. To investigate this, we fit a generalized linear mixed effects model with random intercepts for postnatal age.

We fit both generalized linear and generalized linear mixed models using the package glmmTMB in the R statistical computing environment (Brooks *et al.* 2017; https://www.r-project.org/). We based inferences on a combination of model coefficient estimates (*β*), *P*-values from *post* *hoc* multiple comparisons tests corrected via Tukey’s method, predicted marginal (fixed effects models) and conditional (mixed effects models) effects and their 95% confidence intervals (Onukwugha *et al.* 2015), and standardized effect sizes (*d*).

## Results

### Diffuse TBI suppressed acute neurological reflexes

**Figure 2:**
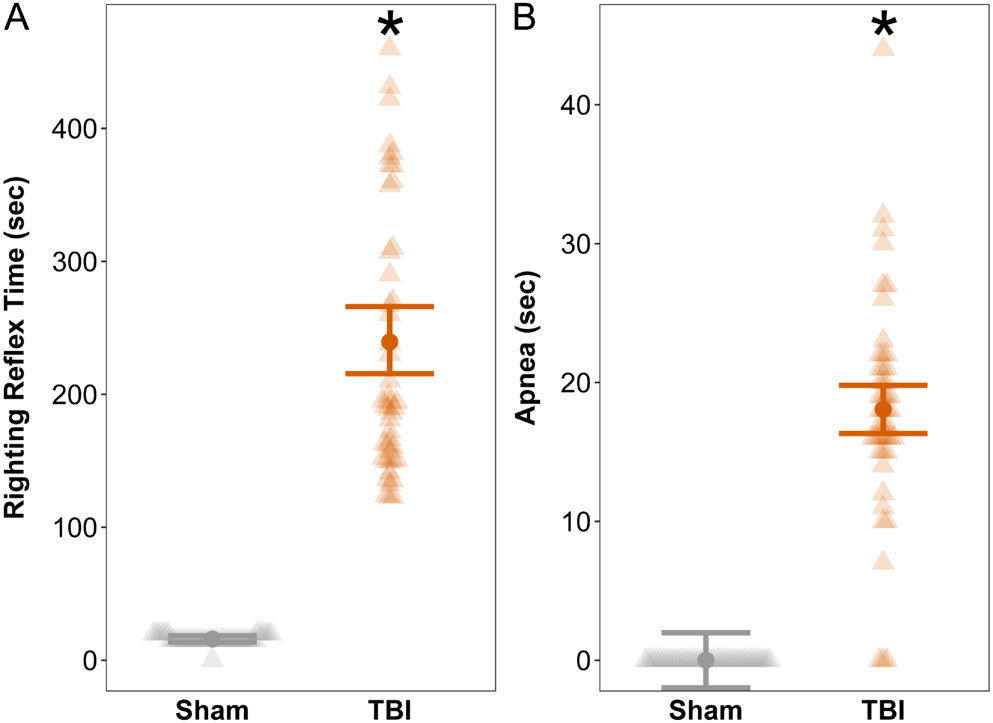
Diffuse TBI suppressed acute neurological reflexes. Righting reflex time, time from the initial impact until the rat spontaneously righted itself from a supine position, and apnea time were measured as indicators of injury severity. Results shown are predicted marginal effects point estimates and 95% confidence intervals from generalized linear models; background triangles denote observed data points. Rats that were subjected to TBI had longer (A) righting reflex times and (B) apnea times compared to uninjured shams. * indicates statistically significant difference (*P* < 0.05).

Rats that were subjected to a diffuse TBI had considerably longer righting reflex times (*β* = 2.71, *P* < 0.001, *d* = 0.35; Fig. 2A) and apnea times (*β* = 18.06, *P* < 0.001, *d* = 3.05; Fig. 2B) than did uninjured shams.

### Brain-injured rats had a lower terminal body weight at 18 DPI compared to shams

**Figure 3:**
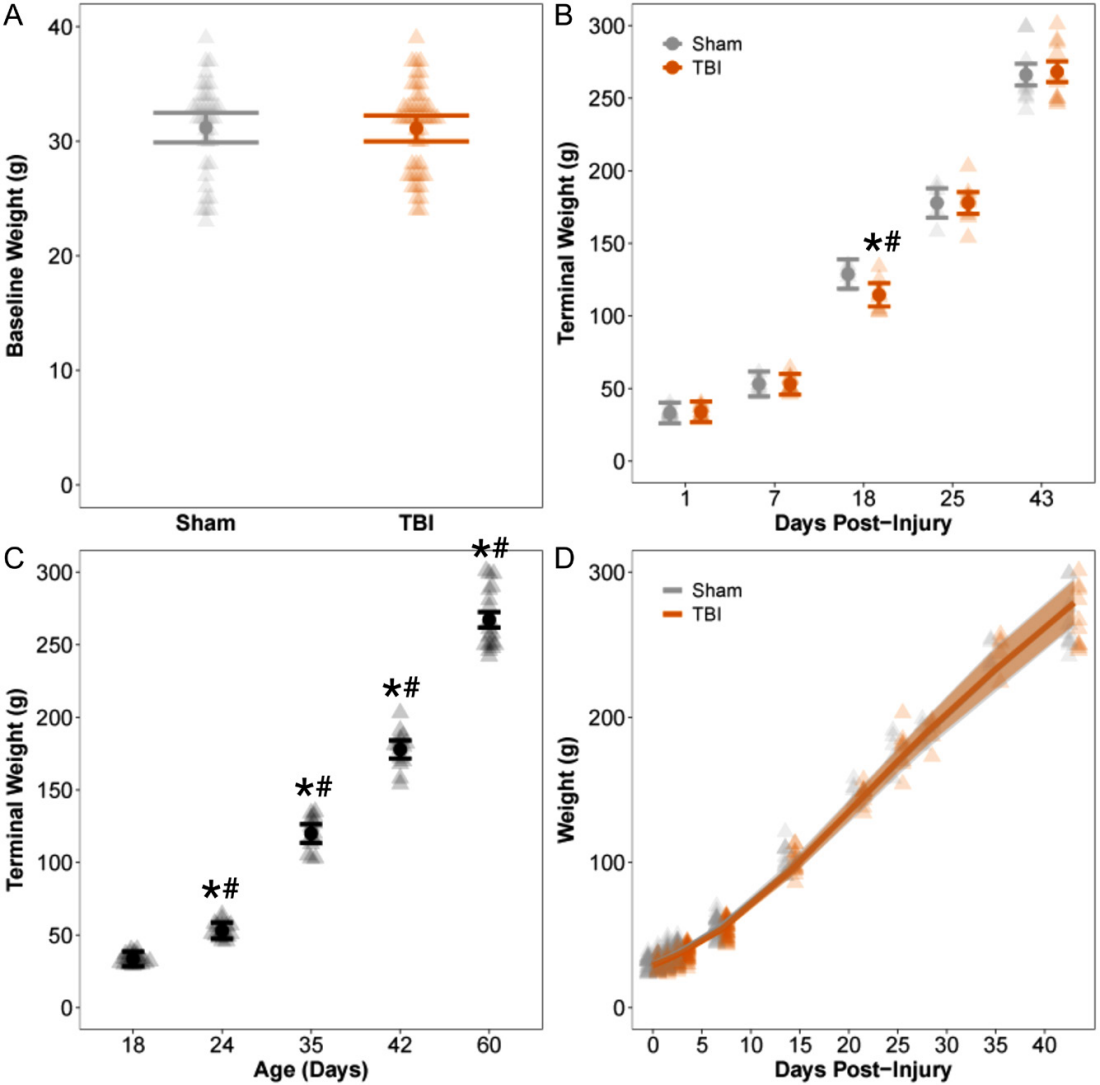
Brain-injured rats had lower body weights at 18 days post injury (DPI) compared to sham. Results shown as predicted marginal effects point estimates and 95% confidence intervals; background triangles denote observed data points. (A) There were no statistically significant differences in baseline body weights taken prior to surgery/injury. (B) There were no brain injury-induced differences in terminal body weights of rats at 1, 7, 25, or 43 days post injury (DPI). * indicates *P* < 0.05 compared to sham. However, brain-injured rats weighed significantly less than uninjured shams at 18 DPI. (C) Terminal body weights increased over time with animal age. Compared to postnatal day 18, rats had higher body weights at postnatal days 24, 35, 42, and 60. * indicates *P* < 0.05 compared to postnatal day 18. (D) Terminal body weight increased nonlinearly over time with animal age at a mean rate of *λ* = 1.19, or 19% per day (95% CI = 17–22%). * indicates statistically significant difference (*P* < 0.05); # indicates biologically significant difference (*d* ≥ 1.0).

There were no statistically significant differences in the surgery weights of rats assigned to the sham group compared to rats assigned to the TBI group (*β* = −0.07, *P* = 0.94, *d* = 0.02; Fig. 3A). There were no statistically significant brain injury-induced differences in terminal body weights of rats at 1 DPI (mean ∆ = 0.6, *P* = 0.91, *d* = 0.05; Fig. 3B), 7 DPI (mean ∆ = 0.14, *P* = 0.98, *d* = 0.01; Fig. 3B), 25 DPI (mean ∆ = 0.09, *P* = 0.99, *d* = 0.01; Fig. 3B), or 43 DPI (mean ∆ = 1.98, *P* = 0.71, *d* = 0.17; Fig. 3B); however, brain-injured rats weighed significantly less at 18 DPI compared to uninjured shams (mean ∆ = 14.3, *P* = 0.03, *d* = 1.24; Fig. 3B). Independent of brain injury, terminal body weights increased over time with animal age (Fig. 3C). Compared to postnatal 18 days, rats had significantly higher body weights at postnatal 24 days (mean ∆ = 19.5, *P* < 0.001, *d* = 1.64; Fig. 3C), postnatal 35 days (mean ∆ = 86.4, *P* < 0.001, *d* = 7.30; Fig. 3C), postnatal 42 days (mean ∆ = 144.3, *P* < 0.001, *d* = 12.19; Fig. 3C), and postnatal 60 days (mean ∆ = 233.7, *P* < 0.001, *d* = 19.74; Fig. 3C). We also assessed the growth rates of sham and brain-injured rats. Body weight increased nonlinearly over time with animal age at an average rate of *λ* = 1.19, or approximately 19% per day (95% CI = 17–22%; Fig. 3D); body weight changes over time (growth rate) did not differ between sham and injured rats (*β* = −0.02, *P* = 0.69).

### GH levels increased with age and were altered by TBI

**Figure 4:**
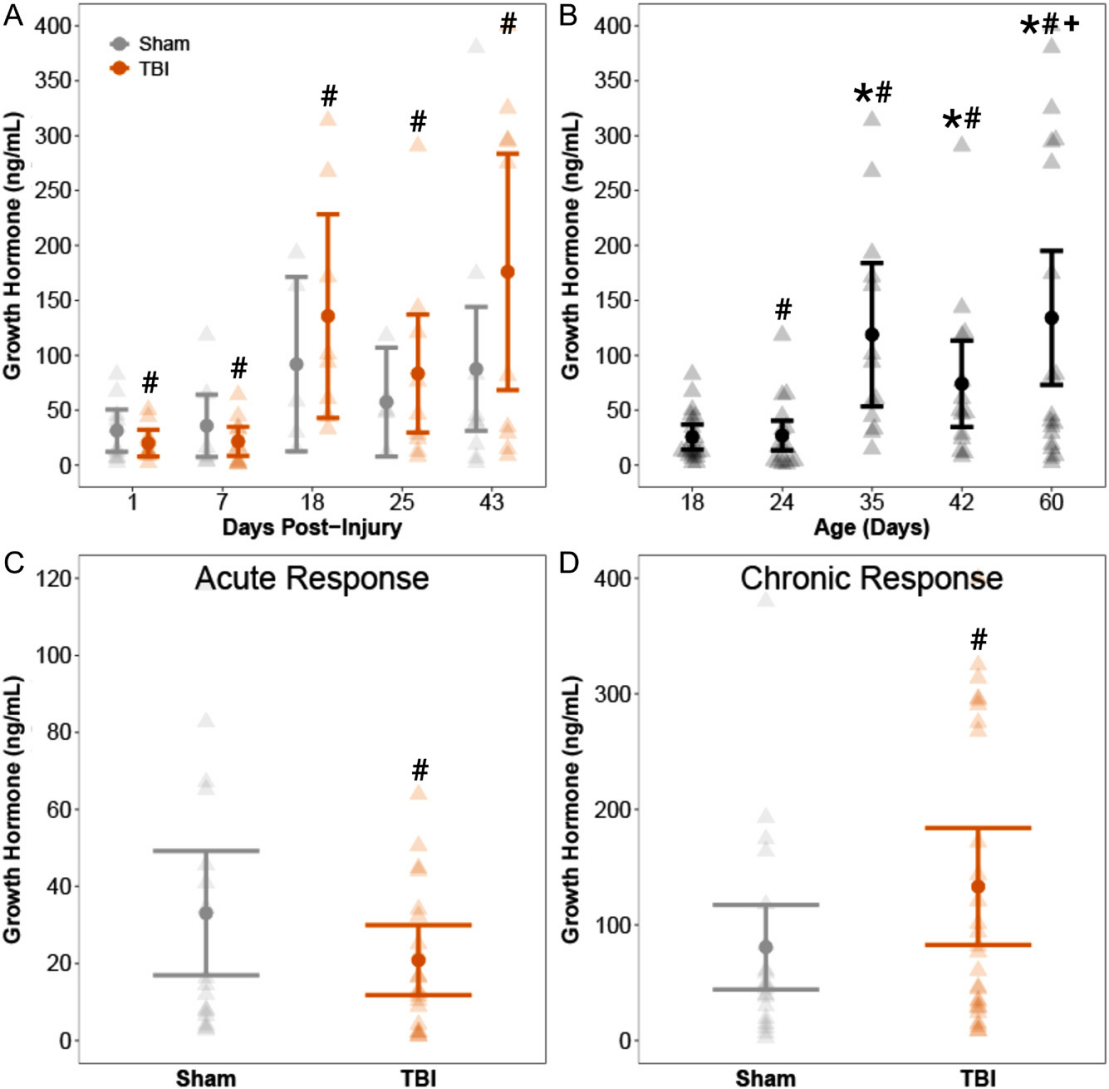
Growth hormone (GH) levels increased over time with animal age and were altered by TBI. Results shown as predicted marginal effects point estimates and 95% confidence intervals; background triangles denote observed data points. (A) There were no differences in plasma GH levels between sham and TBI rats at any terminal time point. (B) Compared to postnatal day 18, GH levels were comparable at postnatal day 24 but were significantly elevated at postnatal days 35, 42, and 60. GH levels at postnatal day 60 were also elevated compared to postnatal day 24. * indicates *P* < 0.05 compared to postnatal day 18; + indicates *P* < 0.05 compared to postnatal day 24. (C–D) We further assessed TBI-induced changes during the acute period (1 and 7 DPI) and the chronic period (18, 25, and 43 DPI). Compared to shams, GH levels were lower in the TBI group during the acute period, but higher in the TBI group during the chronic period. Those differences were not statistically significant but had very large effect sizes indicating biological significance. * indicates statistically significant difference (*P* < 0.05); # indicates biologically significant difference (*d* ≥ 1.0).

Although there were no statistically significant differences in plasma GH levels between sham and TBI rats within any terminal time point, the standardized effect sizes for all differences within every time point were large: 1 DPI (mean ∆ = 11.4, *P* = 0.33, *d* = 11.54; Fig. 4A), 7 DPI (mean ∆ = 14.2, *P* = 0.38, *d* = 14.38), 18 DPI (mean ∆ = 43.8, *P* = 0.48, *d* = 44.42); 25 DPI (mean ∆ = 25.9, *P* = 0.49, *d* = 26.26), and 43 DPI (mean ∆ = 88.5, *P* = 0.16, *d* = 89.72). Compared to postnatal 18 days, GH levels were comparable at postnatal 24 days (mean ∆ = 1.3, *P* = 0.88, *d* = 1.29; Fig. 4B) but were significantly elevated at postnatal 35 days (mean ∆ = 93.179, *P* = 0.006, *d* = 92.24), postnatal 42 days (mean ∆ = 48.44, *P* = 0.02, *d* = 47.95), and postnatal 60 days (mean ∆ = 108.42, *P* < 0.001, *d* = 107.32). GH levels at postnatal 60 days were also significantly elevated compared to postnatal 24 days (mean ∆ = 107.1, *P* = 0.01, *d* = 106.03; Fig. 4B). We further assessed TBI-induced changes during the acute period (1 and 7 DPI) and the chronic period (18, 25, and 43 DPI). Compared to shams, GH levels were lower in the TBI group during the acute period (*β* = −12.22, *P* = 0.196, *d* = 12.3; Fig. 4C) but higher in the TBI group during the chronic period (*β* = 52.41, *P* = 0.10, *d* = 52.1; Fig. 4D); although not statistically significant, the standardized effect sizes for those differences were large.

### The number of somatostatin neurons did not substantially differ between brain-injured and sham rats

**Figure 5:**
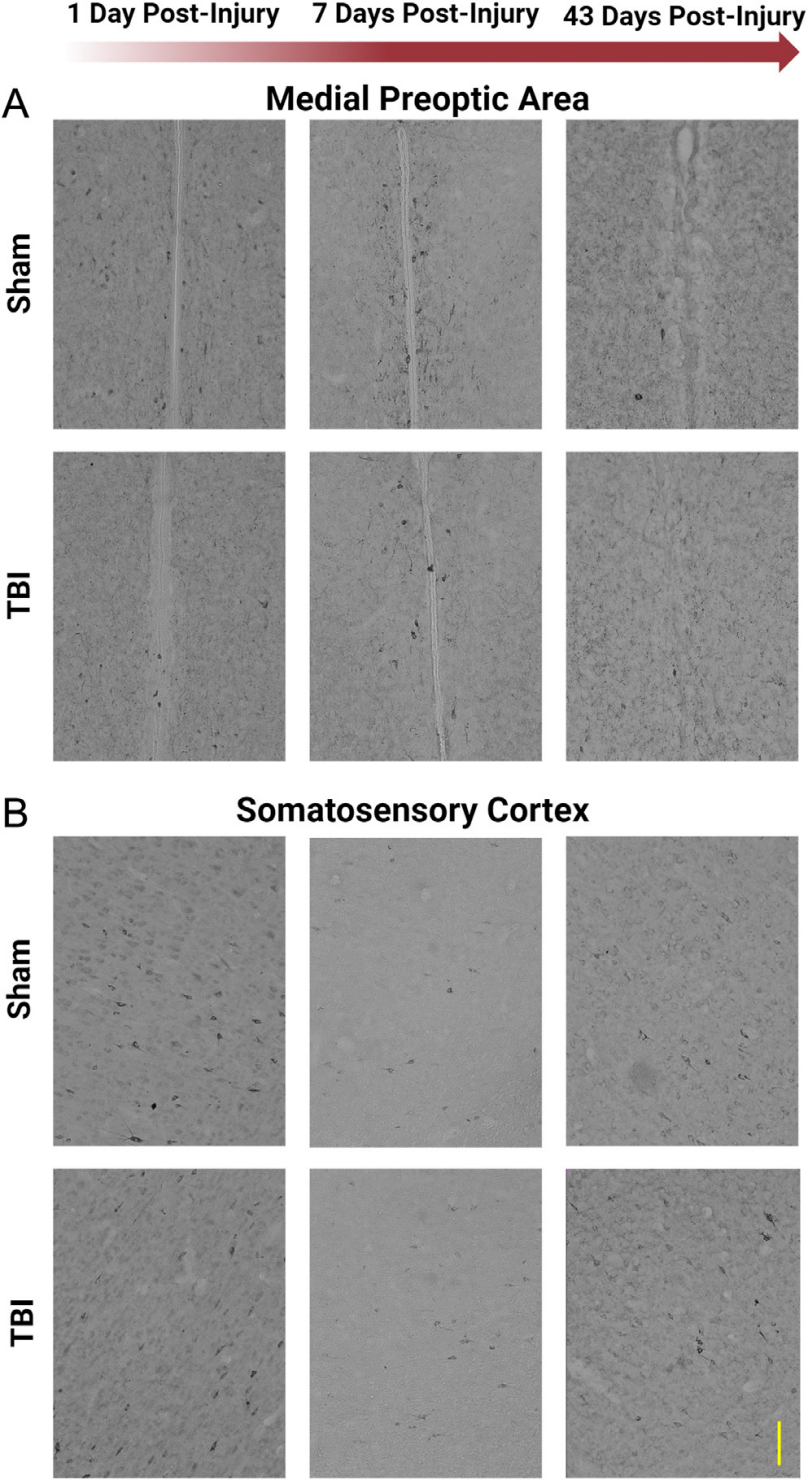
Representative images of somatostatin neurons in the medial preoptic area and somatosensory cortex. Somatostatin-stained neurons in the (A) medial preoptic area and (B) the somatosensory cortex from rats subjected to diffuse TBI or a control sham surgery. Representative images were taken at 1, 7, and 43 days post injury. Scale bar = 100 µm.

**Figure 6:**
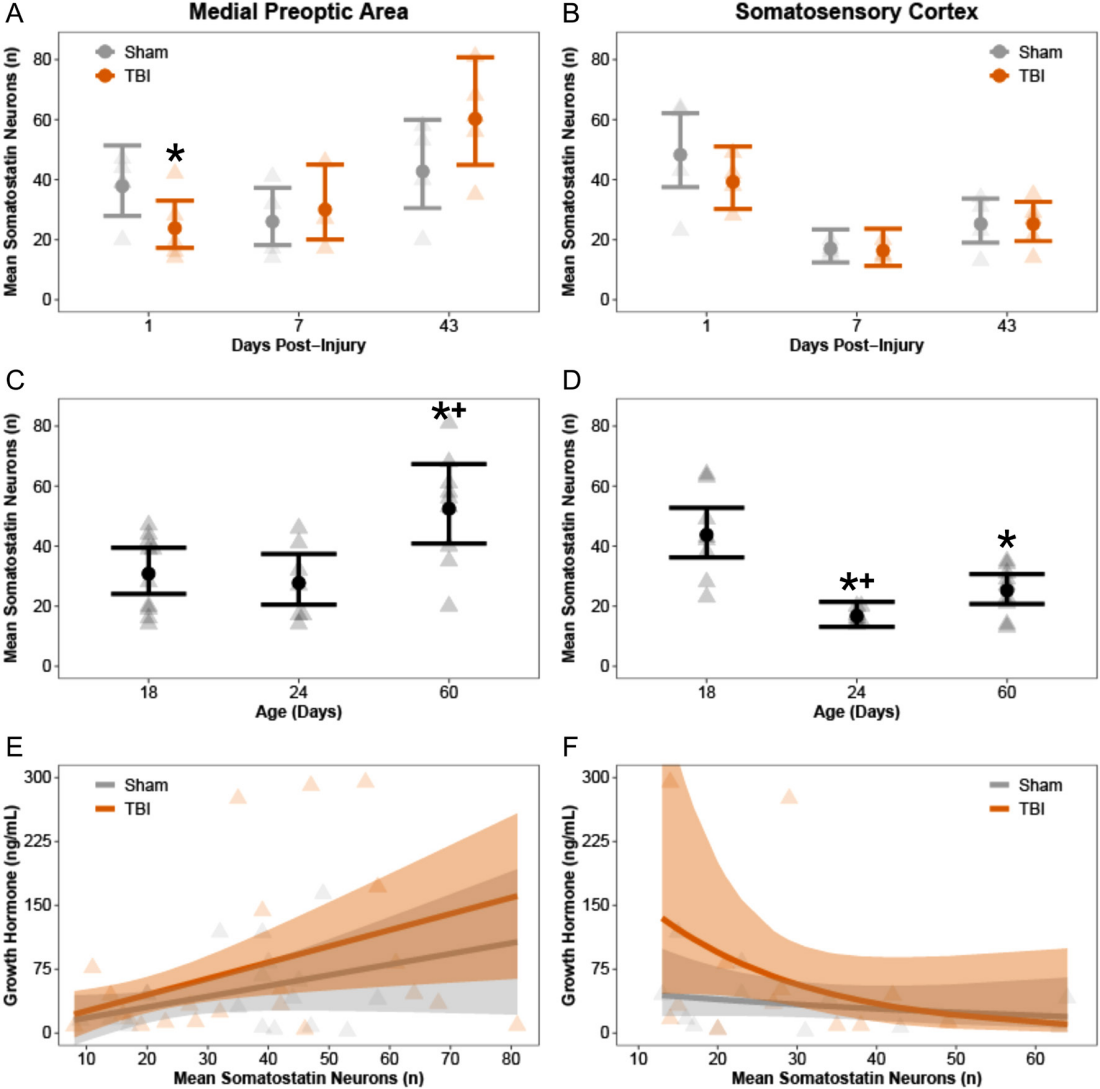
Mean number of somatostatin neurons predicted growth hormone (GH) levels. Changes in somatostatin neurons over time and whether neurons predicted GH levels were assessed in the (A, C, E) medial preoptic area and (B, D, F) somatosensory cortex. In both the (A) medial preoptic area and (B) somatosensory cortex, TBI rats had less somatostatin neurons at 1 day post injury (DPI) compared to shams, whereas no differences existed at 7 or 43 DPI. (C) The mean number of somatostatin neurons increased over time with animal age in the medial preoptic area, whereas (D) the mean number of somatostatin neurons decreased with age in the somatosensory cortex. (E) Irrespective of sham or TBI (i.e. treatment groups combined), the number of neurons was a positive predictor of GH in the medial preoptic area; for every additional neuron, an average of 1.27 ng/mL increase in GH was predicted. (F) In contrast, irrespective of sham or TBI, the number of neurons was not a significant predictor of GH in the somatosensory cortex. * indicates *P* < 0.05 compared to sham; + indicates *P* < 0.05 compared to postnatal day 18.

The mean number of somatostatin neurons in both the medial preoptic area and somatosensory cortex was calculated for each rat (Fig. 5). Although rats subjected to TBI had less somatostatin neurons in the medial preoptic area at 1 DPI compared to shams, the effect size was very small (mean ∆ = 14, *P* = 0.05, *d* = 0.04; Fig. 6A). There were no notable differences in the mean number of somatostatin neurons in the medial preoptic area at 7 DPI (Mean ∆ = 4, *P* = 0.61, *d* = 0.01) or 43 DPI (mean ∆ = 17, *P* = 0.15, *d* = 0.03). Similarly, differences in somatostatin neurons in the somatosensory cortex between sham and TBI rats were not supported at 1 DPI (mean ∆ = 9, *P* = 0.27, *d* = 0.01), 7 DPI (mean ∆ = 1, *P* = 0.87, *d* = 0.001), or 43 DPI (mean ∆ = 0, *P* = 0.99, *d* = 0.00; Fig. 6B). Independent of brain injury, the mean number of somatostatin neurons in the medial preoptic area increased over time with animal age (Fig. 6C); compared to postnatal 18 days, the mean number of somatostatin neurons was comparable at postnatal 24 days (mean ∆ = 3, *P* = 0.86, *d* = 0.01) but was significantly higher at postnatal 60 days (mean ∆ = 21, *P* = 0.02, *d* = 0.07), and the mean number of somatostatin neurons at postnatal 60 days was higher than at postnatal 24 days (Mean ∆ = 24, *P* = 0.01, *d* = 0.08). In contrast, the mean number of somatostatin neurons in the somatosensory cortex decreased over time with animal age (Fig. 6D); compared to postnatal 18 days, the mean number of somatostatin neurons was significantly lower at postnatal 24 days (mean ∆ = 27, *P* < 0.0001, *d* = 0.05) and postnatal 60 days (mean ∆ = 19, *P* = 0.0002, *d* = 0.03), and the mean number of neurons at postnatal 24 days was lower than at postnatal 60 days (mean ∆ = 8, *P* = 0.03, *d* = 0.02).

### Mean number of somatostatin neurons positively predicted GH levels in the medial preoptic area

We found no statistically significant difference between sham and TBI for the relationship between somatostatin neurons in the medial preoptic area and growth hormone levels (*β* = 0.65; *P* = 0.55; Fig. 6E). However, irrespective of sham or TBI (i.e. treatment groups combined), the number of somatostatin neurons in the medial preoptic area was a positive predictor of growth hormone levels; for every additional neuron, an average of 1.27 ng/mL (95% CI = 0.21–2.72) increase in growth hormone was predicted. In contrast, no statistically significant difference existed between sham and TBI for the relationship between somatostatin neurons in the somatosensory cortex and growth hormone levels (*β* = −0.03; *P* = 0.33), and the number of somatostatin neurons in the somatosensory cortex was not a predictor of growth hormone levels (*β* = −0.02; *P* = 0.31; Fig. 6F).

## Discussion

It has been widely shown through clinical and translational studies that TBI can lead to endocrinopathies. In adults, GHD via disruption of the GH axis is the most common endocrinopathy following a TBI (Agha *et al.* 2007, Kgosidialwa *et al.* 2019). In juveniles, however, less is known about the progression of post-TBI endocrine function (Wexler *et al.* 2023). Indeed, both retrospective and prospective clinical studies have found variable rates of GHD in pediatric populations (Aimaretti *et al.* 2004, Auble *et al.* 2014, Briet *et al.* 2019). Here, we modeled diffuse brain injury in adolescence by subjecting juvenile rats to diffuse brain injury and assessed TBI-induced pathology and function of the GH axis.

To the best of our knowledge, the present study is the first to provide longitudinal data on the growth rates of juvenile rats following a diffuse brain injury. We observed that juvenile rats gained weight nonlinearly over time with animal age, with similar growth rates between TBI and sham groups. Although growth rates were similar between TBI and sham rats, we found that brain injured rats weighed significantly less than shams at 18 DPI. Thus, despite TBI having no effect on overall growth rates or the terminal body weight of rats at chronic time points, a significant weight difference was observed at postnatal day 35 (postnatal day 17 at injury + 18 DPI = postnatal day 35). Considerable variation exists in the onset of puberty in male rats, but postnatal day 35 is typically when the onset of puberty occurs (Sengupta 2013, Venugopal 2019). Additionally, preputial separation, a physiological marker of puberty, is typically observed near postnatal day 35 in the male rat (Korenbrot *et al.* 1977). Moreover, as we have previously described, pediatric TBI and injury-induced endocrine disruptions have been indicated as a potential cause of altered puberty (Ortiz *et al.* 2019, 2022). Accordingly, the marked weight difference we observed at postnatal day 35 may be explained as a pubertal delay from acute TBI pathophysiology.

Following both TBI and sham conditions, GH levels increased over time with animal age. Although longitudinal measurements of post-TBI GH levels have yet to be recorded in rats, many clinical studies provide ancillary support for the general trend that we observed. Specifically, GH production tends to vastly increase during puberty, and then gradually declines during adulthood (Rose *et al.* 1991, Saenger 2003). In the current study, TBI substantially impacted GH production. GH levels were lower in the acute period (1 and 7 DPI) and higher in the chronic period (18, 25, and 43 DPI) in rats that sustained a TBI compared to uninjured shams. Despite these results not being statistically significant, the standardized effect sizes were large, which conveys a biologically significant finding. Thus, GH production may be differentially altered in the acute postinjury period compared to the chronic postinjury period and additional studies are warranted to investigate alterations to the GH axis in response to TBI.

Much of the current literature indicates that GHD represents the majority of hormonal changes observed in the acute postinjury period (Gasco *et al.* 2021). The acute decrease in GH observed in this study is likely indicative of the adaptive response to pathological changes in the hypothalamus following TBI (Gasco *et al.* 2021). In contrast, in the chronic postinjury period, rats that sustained a TBI had higher GH levels compared to shams. In other words, a clear GH overshoot was apparent following acute GHD. Transient GHD following TBI has previously been described in clinical studies in adults (Aimaretti *et al.* 2004, Aimaretti *et al.* 2005). Further, previous studies have found that GH levels in the chronic post-injury period were significantly lower in adult rats 2 months after controlled cortical impact (CCI) compared to shams (Kasturi & Stein 2009). The discrepancy between these data and that of the current study could be due to a variety of factors, including the age at which the rats were injured, and the overt cell death associated with a focal injury model (Pleasant *et al.* 2011). Further, since GH has also been found to play an important role in neurogenesis following injury, this chronic GH increase could potentially be evidence of a strong, neurogenerative injury response apparent in adolescence (Scheepens *et al.* 1999, Aberg *et al.* 2009).

At 1 DPI, the number of somatostatin neurons in the medial preoptic area of the hypothalamus acutely decreased in rats subjected to diffuse TBI compared to uninjured shams. This decrease in number of somatostatin neurons at 1 DPI directly coincides with the acute decrease in GH that we observed in brain injured rats. Although the number of somatostatin neurons in the somatosensory cortex also decreased at 1 DPI, this change was not significant. It is plausible that the acute decrease in neurons observed in the current study is not specific to somatostatin neurons but is a result of a general loss of neurons or neuronal complexity in the acute phase post TBI (Akamatsu & Hanafy 2020).

Interestingly, we found changes in somatostatin neurons over time with animal age were brain region-specific. Somatostatin neuron number increased over time with animal age in the medial preoptic nucleus of the hypothalamus. In contrast, the number of somatostatin neurons in the somatosensory cortex decreased over time with animal age. We have previously observed significant neuropathology, identified by hyperintense deposition of argyrophilic reaction product (amino-cupric silver histochemical technique), in the somatosensory cortex of rats subjected to midline fluid percussion injury (Beitchman *et al.* 2021). This brain region is adjacent to the injury site and is particularly vulnerable to neurodegeneration (Beitchman *et al.* 2021). The spatial distribution of neuron injury and death after fluid percussion injury is primarily noted in the cortex and hippocampus (Hicks *et al.* 1996). Based on the biomechanics of the injury model, the somatosensory cortex may be more vulnerable than the hypothalamus to injury-induced damage in injured rats, as well as juvenile sham rats, due to possible cellular disruption from the control craniectomy.

Overall, the mean number of somatostatin neurons was highest in the medial preoptic nucleus of the hypothalamus of brain injured rats in tissue collected at the chronic 43-day time point. Neurons in the medial paraventricular nucleus of the hypothalamus have altered morphologies following diffuse TBI in adult rats (Rowe *et al.* 2016*a*). We previously observed an increase in the number of branch points and hypothalamic neuronal complexity, which coincided with a decrease in corticosterone production in the chronic postinjury period (Rowe *et al.* 2016*a*); thus, structural changes contributed to functional changes. This is supported by other research that indicated chronic dendritic arborization and hypertrophy distal to the injury site following fluid percussion in the rat (Hoffman *et al.* 2017).

In the medial preoptic area of the hypothalamus, we observed a positive relationship between the number of somatostatin neurons and growth hormone levels in both uninjured sham rats, and rats subjected to diffuse TBI. Presumably, this increase in the number of somatostatin neurons may be indicative of feedback regulation observed as a function of age. Rats subjected to TBI had higher GH and more somatostatin neurons in the chronic period compared to the acute period. Increases in GH levels in the chronic postinjury period may lead to hypersecretion of hypothalamic somatostatin. A clinical study in patients with acromegaly, a condition characterized by chronic excess of GH, showed that patients with acromegaly also have high levels of somatostatin and concluded that excess GH could be a causal factor in somatostatin hypersecretion (Arosio *et al.* 2003). In the somatosensory cortex, we did not detect a relationship between the number of somatostatin neurons and growth hormone levels in either uninjured sham rats or rats subjected to diffuse TBI. Further experiments are needed to investigate this potential brain region-specific relationship between somatostatin neuron number and GH production.

The results of this study should be interpreted in the context of a few limitations. Primarily, other hormonal components of the GH axis, such as growth hormone releasing hormone (GHRH) and insulin-like growth factor 1 (IGF-1), were not measured. Both GHRH and IGF-1 have major contributions to the regulation of GH and therefore are likely to contribute to GHD. Historically, both IGF-1 and GHRH measurements have been employed as diagnostic criteria of GHD (Aimaretti *et al.* 2004, Ibba *et al.* 2020). Thus, it should be considered that the chronic increase we observed in postinjury GH levels, and subsequent increase in somatostatin neurons, are confounded by potential pathological changes in IGF-1 or GHRH secretion. Future studies should be performed with measurements of all GH-axis hormones, providing a more complete understanding of post-TBI pituitary function. We also importantly note that only male rats were used in the present study. The age of sexual maturity in female rats is generally accelerated, so pubertal delays resulting from TBI, if present, should be investigated following a sex-specific timeline post injury. Further, the female hormonal milieu unmistakably differs from that of male rats; therefore, additional studies are warranted to determine if sex is a relevant biological variable in juvenile hypopituitarism with respect to the GH axis.

## Conclusion

Current research indicates that TBI-induced endocrine dysfunction can prolong recovery, but little research has focused on the pediatric population. Our findings help address a deficit in research by investigating endocrine dysfunction in juvenile rodents. We found that diffuse TBI in juvenile rats leads to dysfunction of the GH axis. Understanding TBI-induced alterations in the GH axis may identify therapeutic targets to improve the quality of life of pediatric TBI survivors. We also observed age-specific effects on body weight that may be associated with delayed onset of puberty. These findings support the implementation of screening protocols for pediatric survivors of TBI to identify early disruptions in puberty and endocrine dysfunction. Future clinical and preclinical studies likely would be able to address the limitations described earlier and may provide an even greater understanding of how TBI impacts function of the GH axis in the pediatric population.

## Declaration of interest

The authors declare that they have no conflict of interest that could be perceived as prejudicing the impartiality of the research reported.

## Funding

These experimental studies were funded by the National Institutes of Health http://dx.doi.org/10.13039/100000002 R21NS120022 (RR), the Valley Research Partnership VRP43 223010 (JO, RR), and the Brain Injury Association of America http://dx.doi.org/10.13039/100014906 Seed Grant (JO, RR). ST was supported by the National Institutes of Health http://dx.doi.org/10.13039/100000002 Workforce Inclusion in Neuroscience through Undergraduate Research Experience (WINURE) at Arizona State University http://dx.doi.org/10.13039/100007482. NC and GSM were supported by the University of Colorado http://dx.doi.org/10.13039/100010174 Boulder Undergraduate Research Opportunities Program (UROP) fellowship.

## Author contribution statement

JO assisted with conceptualization, data collection, and led writing of the manuscript. ST, GR, GSM, NC, MM, and TG assisted with experimental design, data collection, and editing the manuscript. SMM led statistical analyses and assisted with reviewing and editing the manuscript. RR was responsible for conceptualization, experimental design, funding acquisition, formal analysis, writing, and reviewing and editing the manuscript.
